# Annual acknowledgement of manuscript reviewers

**DOI:** 10.1186/2049-1891-6-2

**Published:** 2015-02-09

**Authors:** Defa Li

**Affiliations:** China Agriculture University, Beijing, China

## Abstract

The editors of *Journal of Animal Science and Biotechnology* would like to thank all our reviewers who have contributed to the journal in Volume 5 (2014).

**Tomas J Acosta**

Japan

**David Adelson**

Australia

**Juliana Almeida**

United States of America

**Jill Anderson**

United States of America

**Robin Anderson**

United States of America

**Cheryl Ashworth**

United Kingdom

**Jean-Denis Bailly**

France

**Fuller Bazer**

United States of America

**Denise Beaulieu**

Canada

**John Bernard**

United States of America

**Donagh Berry**

Ireland

**Mario Binelli**

Brazil

**John Black**

Australia

**Joaquim Brufau**

Spain

**Luigi Calamari**

Italy

**Ana Calderon de la Barca**

Mexico

**Anthony Capuco**

United States of America

**Eduardo Casas**

United States of America

**You-Tzung Chen**

Taiwan

**Lee I. Chiba**

United States of America

**Mingan Choct**

Australia

**Youngsok Choi**

South Korea

**Iain Clarke**

Australia

**Giuseppe Conte**

Italy

**G Andres Contreras**

United States of America

**Joe Crenshaw**

United States of America

**Thomas Crenshaw**

United States of America

**Dirk Dannenberger**

Germany

**Teresa Davis**

United States of America

**Laura De Martino**

Italy

**Christian Diot**

France

**James Drackley**

United States of America

**Caroline Eozenou**

France

**Kay Faaberg**

United States of America

**Catherine Field**

Canada

**Jeffrey Firkins**

United States of America

**Carolyn Fitzsimmons**

Canada

**Jorge Flores**

United States of America

**William Flowers**

United States of America

**Stephen Ford**

United States of America

**Niamh Forde**

Ireland

**Michel A Fortier**

Canada

**Abigail Fowden**

United Kingdom

**George Foxcroft**

Canada

**Anita Franczak**

Poland

**Rick Funston**

United States of America

**Shaorong Gao**

China

**Dorian Garrick**

United States of America

**Joël Gautron**

France

**Rodney Geisert**

United States of America

**Ilias Giannenas**

Greece

**Sylvie Giger-Reverdin**

France

**Guillermo Giovambattista**

Argentina

**Robert Goodband**

United States of America

**Alison Gosby**

Australia

**Heather Greenlee**

United States of America

**Veronika Halas**

Hungary

**Joshua Hare**

United States of America

**Kenton Hart**

United Kingdom

**Zhixiong He**

Canada

**Jung Min Heo**

South Korea

**Jill Hiney**

United States of America

**Asraf Hossain**

India

**Kazuhiko Imakawa**

Japan

**Robert Ivarie**

United States of America

**Eui-Bae Jeung**

South Korea

**Peng Ji**

United States of America

**Li Jiang**

China

**Chen Jilan**

China

**Greg Johnson**

United States of America

**Hakhyun Ka**

South Korea

**Monika Kaczmarek**

Poland

**Filiz Karadas**

Turkey

**Levent Keskintepe**

United States of America

**Tarek Khalifa**

Greece

**Mahesh Khatri**

United States of America

**Beob Kim**

South Korea

**Inho Kim**

South Korea

**Sung Woo Kim**

United States of America

**Terry Klopfenstein**

United States of America

**Marlon Knights**

United States of America

**Esther Kok**

Netherlands

**Xingen Lei**

United States of America

**Crystal Levesque**

United States of America

**Yan Li**

United States of America

**Changxi Li**

Canada

**Fenge Li**

China

**Ming Li**

United States of America

**Wen Li**

United States of America

**Yanhong Liu**

United States of America

**Jian-Xin Liu**

China

**Li Liu**

China

**Ting Lu**

United States of America

**Robert Lucito**

United States of America

**Rod Mackie**

United States of America

**Kasey Maddock Carlin**

United States of America

**Isabelle Mansuy**

Switzerland

**Milan Marounek**

Czech Republic

**Matthew McCabe**

Ireland

**Phillip Miller**

United States of America

**Byungrok Min**

United States of America

**J. Ernest Minton**

United States of America

**Kamal Mjoun**

United States of America

**Noura Mohamed Salih**

Sudan

**Qinghua Nie**

China

**Akbar Nikkhah**

Iran

**Martin Nyachoti**

Canada

**Jack Odle**

United States of America

**Hakan Oeztuerk**

Turkey

**Padraig O’Kiely**

Ireland

**Gunilla Olivecrona**

Sweden

**Christopher O'Neill**

Australia

**Troy Ott**

United States of America

**Thomas Overton**

United States of America

**Fred Owens**

United States of America

**Donald Palmquist**

United States of America

**Volkmar Passoth**

Sweden

**John Patience**

United States of America

**Amlan Patra**

India

**Zoran Pavlovic**

Serbia

**Olli Peltoniemi**

Finland

**Rosa Maria Pereira**

Portugal

**Christiane Pfarrer**

Germany

**Thonart Philippe**

Belgium

**Karina Pierce**

Ireland

**Rolf Prade**

United States of America

**Katy Proudfoot**

United States of America

**Gerardo Quiroz-Rocha,**

Mexico

**Lawrence Reynolds**

United States of America

**Xavier Rollin**

Belgium

**Sina Safayi**

United States of America

**Olivier Sandra**

France

**Andrew Schally**

United States of America

**Leonardo A Sechi**

Italy

**Anshan Shan**

China

**Zhentao Sheng,**

United States of America

**Mathieu Silberberg**

France

**Roberto Silva**

Brazil

**Panagiotis Simitzis**

Greece

**Raj Kumar Singh**

India

**Dariusz J. Skarzynski**

Poland

**Stephen Smith**

United States of America

**Tamas Somfai**

Japan

**Chad Stahl**

United States of America

**Agata Stanek**

Poland

**Kim Stanford**

Canada

**Hans Stein**

United States of America

**Aijun Sun**

United States of America

**Lakshmi Sunkara**

United States of America

**Sylwester Swiatkiewicz**

Poland

**Kiyoshi Tajima**

Japan

**Fuyuhiko Tamanoi**

United States of America

**Sha Tao**

United States of America

**Narasaraju Teluguakula**

United States of America

**Robert Thaler**

United States of America

**Leo Timms**

United States of America

**Mia Torchetti**

United States of America

**Yutaka Uyeno**

Japan

**Christof Van Poucke**

Belgium

**Trygve Veum**

United States of America

**Kimberly Vonnahme**

United States of America

**Shuichi Wada**

Japan

**Jacqueline Wallace**

United Kingdom

**John Wallace**

United Kingdom

**Ying Wang**

United States of America

**Xin Wang**

United States of America

**Xiaoqiu Wang**

United States of America

**Kevin Wanner**

United States of America

**Malcolm Watford**

United States of America

**Thomas West**

United States of America

**Niki Whitley**

United States of America

**Gu Xianhong**

China

**Sumei Yan**

China

**Ning Yang**

China

**Wenzhu Yang**

Canada

**Ming Yang**

Canada

**Jingdong Yin**

China

**Yulong Yin**

China

**Peiqiang Yu**

Canada

**Lidan Zhao**

United States of America

**Lech Zwierzchowski**

Poland

